# Editorial: The global threat of carbapenem-resistant gram-negative bacteria

**DOI:** 10.3389/fcimb.2022.974268

**Published:** 2022-08-01

**Authors:** Milena Dropa, Ziad Daoud

**Affiliations:** ^1^ University of São Paulo, Public Health School, Environmental Health Department, Environmental Microbiology and Antimicrobial Resistance Laboratory (MicroRes), São Paulo, Brazil; ^2^ College of Medicine, Central Michigan University, and Department of Clinical Microbiology, Michigan Health Clinics, Saginaw, MI, United States

**Keywords:** carbapenem-resistance, carbapenemase, metallo-β-lactamase, antimicrobial activity, infection control

Antimicrobial resistance (AMR) in bacteria is a public health complex issue, accelerated by the inadequate use of antibiotics and driven by many interconnected factors. It is far from being restricted to clinical settings, considering antimicrobial compounds are used in livestock and agriculture for several non-clinical reasons. Carbapenem-resistant Gram-negative bacteria are the most critical microorganisms listed by the World Health Organization (WHO) as the priority pathogens that pose a great threat to human health. They can cause severe and often deadly infections, as well as they carry resistance-encoding genes that can be easily spread among bacteria, especially at (but not restricted to) environments where antibiotic selective pressure is applied. Treatment options for infections caused by carbapenem-resistant bacteria are scarce. In many cases, they are also resistant to aminoglycosides, polymyxins, and tigecycline, and the alternatives end up being the use of newer antibiotics and synergistic combinations. Although carbapenems are mostly used in the human clinical practice, reports of carbapenemase-producing bacteria isolated from animal and environmental sources suggest that the prevention of their spread urges for a One Health approach. Surveillance of clones and resistance genetic determinants can lead to an adequate infection treatment in a hospital environment, as well as it can avoid the spread of resistance if it is extended to other fields, like hospital effluents, sewage treatment plants and animal husbandry sources.

This editorial aims to resume the main content of the 25 articles published in the Research Topic, for which the objective was to provide peer reviewed articles on the carbapenem resistance theme, including surveillance studies conducted in hospitals, communities and the environment, the description and characterization of new carbapenem-resistance mechanisms, newly discovered treatment options or detection methods, and the characterization of endemic or emerging clones.

Antimicrobial activity and novel therapeutic strategies were extensively explored by several manuscripts. Petropoulou et al. analyzed the *in vitro* activity of the combination sulbactam-durlobactam (SD) against 190 carbapenem-resistant *Acinetobacter baumannii* isolates from 11 Greek hospitals. Durlobactam restored sulbactam’s activity against most isolates, and the addition of imipenem further lowered SD’s MIC_90_ by one two-fold dilution. Another promising combination therapy against multidrug resistant (MDR) *A. baumannii*, analyzed by Li et al., is berberine hydrochloride (BBH) with sulbactam, ciprofloxacin, tigecycline or meropenem. *In vitro* results showed a dramatic increase of susceptibility or even reverse of resistance for these antibiotics, and *in vivo* studies showed a stronger antimicrobial activity of the combination BBH plus sulbactam, when compared to monotherapy. Also, the study demonstrates that BBH binds to the AdeB transporter protein, reducing the extrusion of antibiotics by the AdeABC pump. A third study targeting MDR *A. baumannii*, by Côrtes et al., proposed a new DNA aptamer for the rapid identification and potential blocking of *A. baumannii* functions. The aptamer A01, selected and identified by an in-house whole-cell SELEX-based method, showed a significant biding affinity to *A. baumannii*, although for now the time-kill assay did not show an effect on bacterial growth.

The *in vitro* activity of MRX-8, a novel polymyxin analogue in development for the treatment of infections caused by MDR Gram-negative pathogens, was evaluated by Wu et al. against several *Enterobacterales*, non-fermenter bacilli and *Haemophilus* spp. clinical isolates (n=765) from 52 hospitals at 20 locations in China. According to the study literature review, the promising drug is more active and less nephrotoxic than other polymyxins. For all microorganisms, including carbapenem-resistant *E. coli* (CR-Eco) and *K. pneumoniae* (CR-Kpn), MICs for MRX-8 were lower than MICs for polymyxin B and colistin, as well as MRX-8, polymyxin B and colistin showed good activity against CR *P. aeruginosa* (CR-Pae) and CR *A. baumannii* (CR-Aba), showing its potential as a valuable therapeutic option. A similar study, conducted by Yu et al., evaluated bloodstream-infection isolates from several hospitals located at East and Central China, comparing the *in vitro* activity of ceftazidime-avibactam (CZA) and aztreonam-avibactam (AZA) against CR-Eco, CR-Kpn and CR-Pae. Authors concluded that AZA presented superior activity against CR-Eco and CR-Kpn, while CZA showed better effect against CR-Pae. Wang et al. evaluated the *in vitro* AZA and CZA activity in combination with auranofin (AUR), an antirheumatic metallodrug that prevents metallo-β-lactamase (MBL) activity *via* displacement of Zn(II) cofactors from their active sites, against metallo-β-lactamase (MBL)-producing clinical *Enterobacterales*. Authors concluded that AUR can both potentiate AZA and CZA *in vitro* activities against MBL producers and restore AZA activity against AZA resistant mutant strains. Also focusing on the urgency of novel antimicrobials and combinations against carbapenem-resistant gram-negative bacteria, Serral et al. generated and refined a metabolic network for a CR-Kpn strain (Kp13) and carried out topological-based analyses to find likely exploitable pathways to develop novel antimicrobials. The study identifies previously recognized pathways and shows the possibility of targeting cellular functions like the production of lipoate, trehalose, glycine, betaine and flavin, as well as the salvaging of methionine.

Han et al. studied the development of CZA and tigecycline (TGC) resistance in a CR-Kpn strain belonging to sequence type 4496, even without exposure to CZA. The strain was isolated three times from a urinary tract infection, in a two-month period, from a patient exposed to β-lactam and TGC treatments. The third isolate (XDX51) developed CZA and TGC resistance. Whole genome sequencing analyses showed that, when compared to the previous isolates (XDX16 and XDX31), XDX51 carried double copies of *bla*_KPC-2_ in a 108kb IncFII plasmid and exhibited upregulation of AcrAB-TolC efflux pump due to *ramR* gene interruption. Also, there was LPS changes caused by *wbbl* gene interruption that contributed to the TGC resistance. On Cardile et al. study, the gut microbiota profile of liver transplant patients colonized with CR-Kpn was regularly monitored for one year after transplant. Antibiotic prophylaxis was divided into standard or targeted, the last defined as a MDR antibiogram-based ATB prophylaxis. The study concludes that both treatment courses do not affect liver transplant outcomes or the risk of intestinal bacterial translocation, and that the gut microbiota had an increase on beneficial and reduction on harmful bacteria species after the standard prophylaxis.

On a perspective study, Tenover discusses the reluctance of laboratories and key guidelines to use molecular tests as tools for the development of therapeutic strategies for CR bacterial infections. The manuscript highlights the importance of differentiating among the Ambler classes of beta-lactamases, especially when using newer antimicrobial combinations in infections therapeutical approaches. On the other hand, the identification of resistance genes coupled with bacterial identification direct from blood culture bottles or in syndromic panels to predict phenotypic susceptibility is still in discussion, but it is an approach that can be very useful for the guidance of treatment when linked to local epidemiology. A parallel matched case-control study carried out by Hoo et al. analyzed the predictors and outcomes of healthcare-associated infections (HAIs) by carbapenem-nonsusceptible *Enterobacterales* at two hospitals from Singapore. Authors concluded that appropriate management of deep-seated infections, and enhancement of antimicrobial stewardship strategies to prevent carbapenem exposure may be useful in reducing the risk of CnSE-HAIs. Corroborating Tenover‘s study, they also suggest that “efforts should be made to improve antimicrobial therapy in patients, possibly through use of rapid resistance diagnostics and clinical prediction tools to identify patients with the greatest risk for CnSE-HAIs, improving their outcomes”. An antimicrobial stewardship program using a carbapenem-sparing strategy was evaluated by Masri et al. in a retrospective study, with regards to the use of carbapenem before and after the program implementation at a tertiary care center in Lebanon. The appropriateness of empirical and targeted therapy prescriptions increased, and pharmacists’ interventions significantly increased concerning the duration of therapy, dose adjustment, de-escalation to a narrower spectrum antibiotic and use of extended infusion. At a tertiary hospital in Germany, Neidhöfer et al. conducted a study in which carbapenemase-encoding bacteria data was combined with patient’s demographic and clinical information for each isolate. Multiple regression analyses confirmed the role of age and gender in colonization patterns and indicated a role for ethnicity and domicile. Also, the study concludes that *Acinetobacter baumanni* is frequently introduced into the hospital but efficiently controlled at hospital admission, and that VIM-producing *Pseudomonas aeruginosa* poses an increasing risk of colonization according to the length of hospital stays. Authors suggest that preventive measures would be opportune for patients from selected regions, and that the role of ethnicity needs further studies.

High-risk carbapenemase-producing clones of different gram-negative species were described in several studies. In a retrospective cohort study, Hu et al. describe the clinical impact and characteristics of bloodstream infections caused by KPC-producing *P. aeruginosa* ST463 in East China, where it has recently emerged and disseminated, leading to a high mortality rate. Isolates were highly resistant to cephalosporins, monobactam and fluoroquinolones, as well as they were classified as hypervirulent by a larvae model. According to the cohort study, mortality rates were higher for ST463 CR-Pae bloodstream infections, when compared to the non-ST463 cases. WGS data for ST463 strains showed they all carried the virulence genes *exoU* and *exoS*, as well as most of them carried *bla*_KPC-2_ gene. One genome also showed a different *bla*_KPC-2_ environment: a 7-kb composite transposon flanked by two IS*26* elements, located in a 41kb plasmid (pZYPA01). In another study from China, the emergence of a clinical ST63 pandrug-resistant *Acinetobacter pittii* was described by Yang et al. The strain was isolated from the sputum of a patient with acute exacerbations of chronic obstructive pulmonary disease (AECOPD) and carried multiple acquired resistance genes, including *bla*_OXA-58_, inserted into a non-typeable and no-transferable plasmid. Authors highlight that *A. pittii* could represent a reservoir for carbapenem resistance determinants. In Germany, Yao et al. demonstrates the emergence of carbapenem-resistant *Citrobacter* spp. revealed by the results from a genome-based regional surveillance study with 512 isolates of 21 CR *Enterobacterales* from 61 hospitals in three years. The detection rates of KPC, OXA-48-like and MBLs in CR-*Citrobacter* were comparable to that of CR-Kpn, and KPC-2 was mostly located in the MDR pMLST15 IncN plasmid. VIM-1 was also located in IncN plasmids, while OXA-48 carbapenemases were in IncL/M ones. In Argentina, Costa et al. describes the co-occurrence of *bla*_NDM-5_ and *rmtB* genes in a clinical CC354 *E. coli*, recovered from the urine of a female patient who lived in a nursing home and had no traveling history on the previous 12 months. The strain was resistant to all beta-lactams, aminoglycosides and quinolones. WGS showed that the resistance genes were inserted in the same conjugative plasmid, which could be transferred to other high-risk clones.

Huang et al. determined the epidemiological and molecular characteristics of CR-Kpn isolates from a hospital in Southwestern China. ST11 was predominant among the 127 strains, and CZA resistance was higher in children, which authors affirm that could provide new insights for clinical empirical use of antibiotics. Several virulence genes (*mrkD*, *uge*, *kpn*, *fimH*) were detected in most isolates, and IncF plasmids were detected in 57.5% of them. A 359,625 bp IncFII conjugative plasmid carried the *bla*_KPC-2_, together with virulence factors. In the intensive care unit of the same hospital, an outbreak of CR-Kpn was described by Zeng et al. The 51 CR-Kpn clonal isolates also belonged to ST11 and carried *bla*_KPC-2_ and the same virulence genes found by Huang et al.. In addition, authors were able to conjugate *bla*_KPC-2_, indicating the potential horizontal spread of the gene throughout the hospital. The molecular characterization of an IncHI5-like plasmid from an NDM-1-producing *K. pneumoniae* was described by Liu et al. The strain (C39) was isolated from the sputum of an infant patient at Henan, China, and belonged to ST37 and KL 15 serotype. The IncHI5-like plasmid pC39-334kb harbored a MDR region containing a wide variety of resistance genes, including *bla*_NDM-1_ inserted into a ΔIS*Aba125*-*bla*_NDM-1_-*ble*_MBL_-*trpF*-*dsbC* structure. Genomic comparison among IncHI5-like plasmids showed they shared most core genes and carried distinct MDR regions, as well as 80% of them were described in Chinese hospital settings.

The increasing incidence of virulence associated with AMR in *K. pneumoniae* also led to interesting studies. Using *in silico* and *in vitro* analyses, the fitness of RmpA, RmpA2, IucA and IutA proteins was studied by Shankar et al., aiming to propose a reliable marker for clinical identification of hypervirulent CR-Kpn. Aerobactin (*iucA/iutA*) was expressed in high-risk clones that could not express the hypermucoviscous phenotype due to truncated *rmpA/rmpA2*, and *in silico* protein analyses showed that IucA and IutA were more stable than RmpA, also showing less conformational fluctuations on their functional domain. In another *in silico* study, the molecular epidemiology of 521 CR-Kpn genomes from several countries was described by Hu et al., focusing on the presence of *bla*_KPC_ and hypervirulent genotypes (HvKP), including hypercapsule (by *rmpA/rmpA2*), EPS (by *wzy-K1*) and excessive siderophores. Authors conclude that there is a great variety on the virulence gene sets, with hypercapsule presenting the same proportion as excessive siderophores, and that *iucA*, *p-rmpA2* and *p-rmpA* are primary genes inducing Hv-*bla*_KPC_(+)-KP. Also, plasmid analysis shows that IncHI1B carrying virulence genes and IncFII with *bla*_KPC_ constitute the primary combination responsible for Hv-*bla*_KPC_(+)-KP.

The environmental spread of carbapenem-resistance encoding genes was also explored in this Research Topic. Ghiglione et al. characterized the whole genome of two KPC-2-producing strains (*K. quasipneumoniae* subsp. *quasipneumoniae* WW14A and *Enterobacter cloacae* WW19C) isolated from urban sewage at Buenos Aires, Argentina. *bla*_KPC-2_ genes were carried by IncP-6 plasmids which shared 99% similarity within a 72% coverage, but also showed unique regions derived from plasmids from South America, Asia and Europe. Also, WW14A was closely related to a GES-5-producing Taiwanese strain isolated from a hospital wastewater sample in 2015. Authors highlight that IncP-6 plasmids could be an emergent mobile platform for the dissemination of *bla*_KPC_ genes.

Rodriguez et al. studied the occurrence of antimicrobial resistance genes (ARGs), especially *bla*_KPC_, throughout a wastewater treatment plant in Antioquia, Colombia. Quantitative PCR revealed *sul1*, *sul2*, *bla*_KPC_ and *ermB* were the most prevalent ARGs, and a low average reduction of the absolute abundance of these genes in effluent in comparison to influent was observed. Physicochemical and climatological parameters were significantly correlated with the absolute abundance of all ARGs, while dissolved oxygen and precipitation in the sampling day were related to the absolute concentration of *bla*_KPC_ over time.

Finally, a review by Jean et al. resumed the main challenges for limiting the spread of CR and extensively drug resistant (XDR) gram-negative bacteria (GNB) through the clinical scenery. It addresses the global trends of carbapenem resistance among GNB species, resistance mechanisms and case-fatality rates, treatment with novel and/or conventional antimicrobials, diagnostics, screening strategies, *P. aeruginosa* and *A. baumannii* infections in the community setting, economic issues, and infection control policies.

In conclusion, this Research Topic harbors a collection of elegant publications showing the fast and multifaceted carbapenem resistance spread and evolution through several settings and geographical regions. Antimicrobial resistance is pandemic and constitutes a great public health concern that needs continuous surveillance and effective control actions.

## Author contributions

Both authors have provided intellectual contributions to this work, and have approved it for publication.

## Acknowledgments

We are very grateful to all reviewers who dedicated their time and expertise to evaluate the manuscripts.

## Conflict of interest

The authors declare that the research was conducted in the absence of any commercial or financial relationships that could be construed as a potential conflict of interest.

## Publisher’s note

All claims expressed in this article are solely those of the authors and do not necessarily represent those of their affiliated organizations, or those of the publisher, the editors and the reviewers. Any product that may be evaluated in this article, or claim that may be made by its manufacturer, is not guaranteed or endorsed by the publisher.

